# Implementation and Evaluation of Two Nudges in a Hospital’s Electronic Prescribing System to Optimise Cost-Effective Prescribing

**DOI:** 10.3390/healthcare10071233

**Published:** 2022-07-01

**Authors:** Saval Khanal, Kelly Ann Schmidtke, Usman Talat, Asif Sarwar, Ivo Vlaev

**Affiliations:** 1 Warwick Business School, University of Warwick, Scarman Road, Coventry CV4 7AL, UK; 2 Warwick Medical School, University of Warwick, Coventry CV4 7AL, UK; kelly.a.schmidtke@warwick.ac.uk; 3 Behavioral Science, University of Health Science and Pharmacy, St. Louis, MO 63110, USA; 4 Alliance Manchester Business School, University of Manchester, Manchester M13 9PL, UK; usman.talat@manchester.ac.uk; 5 Pharmacy Department, Queen Elizabeth II Hospital, University Hospitals Birmingham NHS Foundation Trust, Birmingham B15 2GW, UK; asif.sarwar@uhb.nhs.uk

**Keywords:** behaviour change technique, nudge, prescription optimisation, prescribing behaviour

## Abstract

Providing healthcare workers with cost information about the medications they prescribe can influence their decisions. The current study aimed to analyse the impact of two nudges that presented cost information to prescribers through a hospital’s electronic prescribing system. The nudges were co-created by the research team: four behavioural scientists and the lead hospital pharmacist. The nudges were rolled out sequentially. The first nudge provided simple cost information (percentage cost-difference between two brands of mesalazine: Asacol^®^ and Octasa^®^). The second nudge provided information about the potential annual cost savings if the cheaper medication were selected across the National Health Service. Neither nudge influenced prescribing. Prescribing of Asacol^®^ and Octasa^®^ at baseline and during the implementation of the first nudge did not differ (at *p* ≥ 0.05), nor was there a difference between the first nudge and second (at *p* ≥ 0.05). Although these nudges were not effective, notable administrative barriers were overcome, which may inform future research. For example, although for legal reasons the cost of medicine cannot be displayed, we were able to present aggregated cost information to the prescribers. Future research could reveal more behavioural factors that facilitate medication optimisation.

## 1. Introduction

Unnecessary spending in one part of a healthcare system constrains other parts, and in so doing, can negatively impact patient care. Over the past decade, medication prescribing costs have risen annually for the National Health Service (NHS) in England. During 2019/20, the cost of medications (at list price) in hospitals accounted for 55.9 percent (£11.7 billion) of the total cost of medications used in the NHS, up from 53.9 percent (£10.2 billion) in 2018/19 [1]. In 2019/20, the total hospital cost (at list price) increased by 14.0 percent from the previous year compared to an increase of just 5.2 percent in primary care [1]. Unless novel interventions occur, hospital expenditure on medicines is likely to continue increasing [2]. Where possible, promoting the use of generic or cheaper brand medicines could reduce the increasing costs while maintaining high-quality patient care.

Interventions aiming to reduce hospital spending often involve removing or restricting clinical access to tests and treatments that are not found to be cost-effective. When clinicians, pharmacists, and patients agree that the potential cost savings warrant constraining choice, such constraints are rarely contested. However, when disagreements arise, nudges could prove beneficial. Nudge interventions involve altering the choice architecture without forbidding or unduly restricting choice. Findings from a systematic review suggest that increasing cost-awareness can influence healthcare professionals’ decision-making without forbidding or restricting options [3]. Based on this insight, Fogarty and colleagues created a nudge intervention to increase cost awareness at the very moment clinicians order a diagnostic test [4]. Their nudge was a simple message displayed on a blood CRP (C-Reactive Protein) assays order form, which stated not only the cost of a single assay (£1) but also the annual hospital expenditure in the previous year (£200,914). The researchers then compared the number of times the test was ordered in the 52 weeks before and after the nudge was applied and found a significant decrease in test orders.

While not explicitly tested in Fogarty’s study, the effectiveness of their simple intervention may partly be explained by the dual-process theories [5]. Dual-process theories suggest that two distinct cognitive systems influence behaviour. The reflective system is more effortful and allows for deeper analyses. The automatic system is typically faster and applies mental shortcuts. While all clinicians know that even low-cost tests cost something (the reflective system), they are unlikely to realise the high aggregated costs if everyone orders those low-cost tests. Presenting clinicians with the high aggregated costs could activate their automatic systems and trigger a more restrained use of tests. The current team’s intervention was planned based on the same insights and dual-process theories.

At the time of our intervention, several brands of mesalazine (also known as mesalamine or 5-aminosalicylic acid) were licensed to treat mild to moderate ulcerative colitis and to maintain remission in ulcerative colitis: Asacol^®^, Mezavant^®^, Octasa^®^, Pentasa^®^, and Salofalk^®^. According to the British National Formulary, there is no evidence to show that any one oral preparation of mesalazine is more effective than another. While delivery characteristics of some brands vary, Asacol^®^ and Octasa^®^ MR tablets have nearly identical delivery profiles and both have Eudragit S enteric coatings [6]. Notably, though, Asacol^®^ is more expensive than Octasa^®^, and many trusts were already trying to persuade clinicians to switch the drugs for suitable patients. Not all patients are suitable, e.g., patients who have a documented adverse reaction to Octasa^®^. Clinicians must assess whether a patient is suitable; when a patient’s condition is stable or improving, continuing to prescribe Asacol^®^ is likely an easier choice [7]. A nudge placed precisely at the time a clinician is making these prescribing decisions, e.g., over an electronic prescribing system, could trigger them to consider the swap. If such a nudge included aggregated price information, it could also help them consider the cost implications.

The current study had two objectives. The first was to assess whether clinicians chose the cheaper brand when presented with simple cost information (percentage difference). The second objective was to assess whether clinicians chose the more affordable brand when presented with aggregated cost information (cost-saving to the NHS system).

## 2. Methods

### 2.1. Study Design and Setting

A cross-sectional study was conducted to evaluate the effectiveness of two nudges to optimise cost-effective prescribing at Queen Elizabeth II Hospital in Birmingham, United Kingdom. The hospital employs approximately 7000 front-line staff (including substantive and bank staff) [8]. The hospital has 1215 patient beds, including 100 critical care beds [9]. Birmingham is the second-largest city in the United Kingdom and the 7th most deprived local authority out of England’s 317 authorities [10]. The nudges were implemented in an electronic prescribing system centrally that all the prescribers in the hospitals use. As the data provided did not contain identifiable information about the prescribers, it is uncertain how many individual prescribers saw the nudges.

### 2.2. Context

The hospital uses an electronic prescribing system called the Prescribing Information and Communication System (PICS). PICS supports full e-prescribing and drug administration, requesting and reporting of laboratory investigations, and clinical observations and assessments. It also allows for extensive order communications, including imaging requests and internal referrals [11]. During our study, each of the two electronic nudges was active for six months. The clinicians saw the nudges while they were in the process of prescribing either medication (Asacol^®^ or Octasa^®^) in outpatient and in-patient settings. Daily prescription data were collected for six months and then converted into weekly data to yield a sufficient number of data points for our time-series analyses.

### 2.3. Development of Interventions

At the outset of our project (pre-protocol), our intervention development was guided by the Behavioural Change Wheel (BCW) methodology and stakeholder engagements [12]. The BCW has previously been used to develop other behavioural interventions for changing patient and health worker behaviour [13,14,15,16]. The initial steps in the BCW direct interventionists to identify a meaningful behavioural problem. Our discussions with key stakeholders, including primary care doctors, secondary care doctors, pharmacists, and behavioural scientists across England helped us identify a meaningful issue to target. For instance, while the prescribing of antibiotics was a meaningful issue, many interventions were already targeting antibiotics. In contrast, interventions targeting Asacol^®^ and Octasa^®^ were less common, and stakeholders believed this issue warranted more attention.

The next steps in the BCW help interventionists to diagnose the reasons for the targeted behavioural problem according to a list of barriers and facilitators described by the Theoretical Domain Framework (TDF) [17,18]. Then it helps interventionists link those reasons to theoretically and empirically supported techniques to change behaviour and to identify a delivery mode. For instance, stakeholders helped us appreciate that NHS clinicians are often unaware of the costs of the medications they prescribe, so techniques designed to increase awareness, e.g., prompts, could be effective. Stakeholders also noted that interventions were more likely to be successful where they appeared at the very moment a clinician makes a prescribing decision, e.g., adding objects to the environment, and so our intervention was implemented through an electronic prescribing system. Stakeholders were uncertain whether such prompts should include simple price comparisons (simple cost information) or the wider cost implications for the NHS system (aggregated cost information), so we evaluated the effectiveness of two prompts.

**Figure 1:**
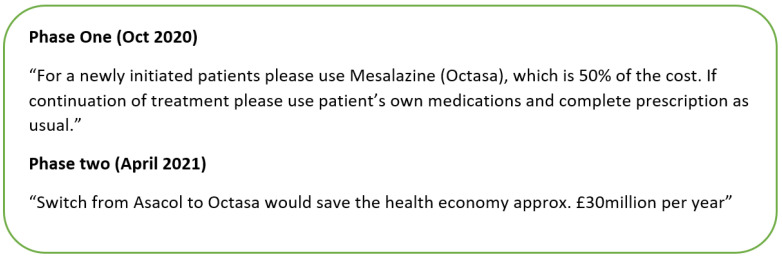
The cost information used as the nudges to promote cost-effective prescribing.

Throughout the intervention development process, the BCW urges interventionists to ensure their intervention is implementable by considering the six key criteria described in the APEASE framework: Affordability, Practicability, Effectiveness and cost-effectiveness, Acceptability, Side-effects and safety, and Equity. For instance, across England’s NHS system, the costs of medicines are individually negotiated by NHS hospital trusts. The hospital trust in this study had an active policy that entailed the cost of medicines that could not be presented directly to the clinicians. Consequently, the research team needed to negotiate a way to present cost information that would not reveal those negotiated costs. Two nudges were agreed upon by the research team, including a lead hospital pharmacist, and approved by the hospital before being implemented. The first nudge provided simple cost information, i.e., the percentage difference between two alternatives. The second nudge provided aggregated cost information, i.e., the potential cost-saving attributed to the medicine switch nationally. Both messages are displayed in Figure 1.

### 2.4. Implementation of the Interventions

**Figure 2:**
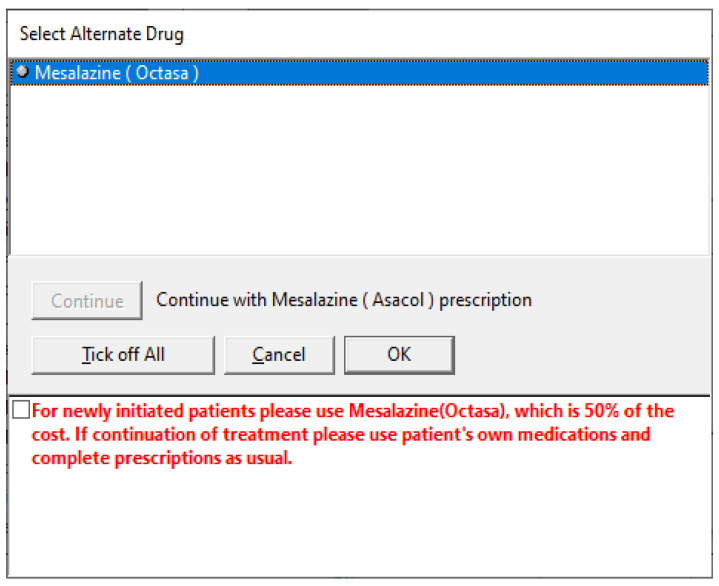
Nudge presented in the PICS prescribing system.

The nudges were implemented through PICS. Prescribing medicine on PICS involves clinicians inputting the relevant patient condition, in this case, “Ulcerative Colitis”. Several approved medication options then appear, and the clinicians can select the appropriate medicine. Before our intervention, the clinicians could directly select Asacol^®^. During our intervention, when clinicians attempted to select Asacol^®^, they then saw one of our nudges and a prompt that needed to be overridden (tick a box) to proceed with prescribing Asacol^®^, see Figure 2. Intervention 1 was implemented for six months, and it was overwritten in the PICS by intervention 2 for the next six months. One should note that both interventions were implemented sequentially; hence, any effect attributed to intervention 2 will also have a sequence effect of the previously implemented intervention 1.

### 2.5. Outcomes and Measurements

The following outcome measures were obtained from the hospital records: (i) the total number of prescriptions of Asacol^®^ per day for 12 months prior to intervention 1 (from 1 October 2019 to 20 September 2020), 6 months during intervention 1 (from 1 October 2020 to 31 March 2021), and 6 months during Intervention 2 (1 April 2021 to 20 September 2021), and (ii) the total number of prescriptions of Octasa^®^ per day for 12 months prior to intervention 1 from 1 October 2019 to 20 September 2020), 6 months during intervention 1 from 1 October 2020 to 31 March 2021), and 6 months during intervention 2 (1 April 2021 to 20 September 2021).

### 2.6. Statistical Analysis

Prescribing data (number of prescriptions) were extracted from the PICS database for both Asacol and Octasa across the pre-Intervention I, post-Intervention I, and post-Intervention II periods. A causal impact analysis was conducted using the Causal Impact package [19] over R-studio Version 1.4.1717. This analysis employed a Bayesian structural time series (BSTM) model, which is a state-space model for time series data [20,21,22,23,24]. The BSTM used in this study modelled the behaviour of the linear predictor and set up prior distributions for unknown quantities in the model for the data before the interventions. The time series components of the BSTM incorporated the trend and seasonality for the number of Asacol and Octasa prescribed by doctors with a basic structural model containing a regression component with a static coefficient. The basic structural time series model used was as follows:

$$y_{t}=\mu_{t}+\tau_{t}+\varepsilon_{t}$$

$$\mu_{t+1}=\mu_{t\;}+w_{t}$$

$$\tau_{t+1\;}=-\overset{S-2}{\underset{s=0}{\sum }}\tau_{t-s\;}+v_{t}$$

where *y_t_* is the number of Asacol/Octasa prescribed for each intervention at a time (week) *t*; *ε_t_*∼$N(0,\sigma_{\varepsilon }^{2})$, *w_t_*∼$N(0,\sigma_{w}^{2})$, and *v_t_*∼$N(0,\sigma_{v}^{2})$ or are iid normal errors. In addition, *μ_t_* is the level or the mean that changes with time and *τ_t_* is the seasonal component with S being the number of seasons. The above equations estimate the post-intervention occurrence difference between the observed time series of the response variable (prescription of Asacol and Octasa) and a simulated (synthetic or forecasted) time series that would have occurred without the intervention [23]. The posterior causal inference works in the following fashion: First, the model is estimated using only the pre-intervention period data. Second, using the estimated model, the forecasts (predictions) of *y_t_* for the post-intervention periods are obtained. Finally, the difference between the forecasted (predicted) values and the actual data (observed values) of *y_t_* during the post-intervention period is interpreted as the causal impact of the interventions.

## 3. Results

### 3.1. Usage of Asacol^®^

#### 3.1.1. Simple Cost Nudge

**Figure 3:**
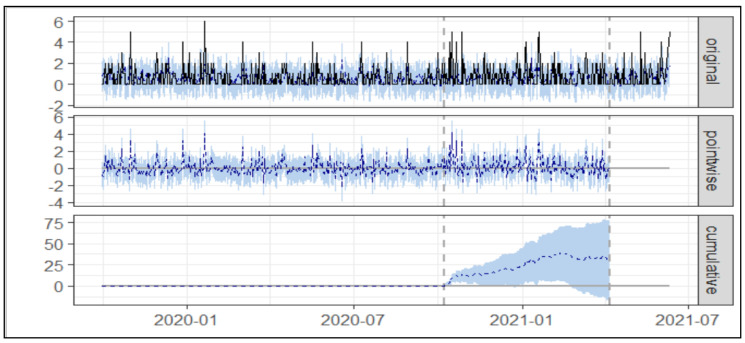
The trend of prescription of Asacol^®^ prior to and during the implementation of intervention 1. Note: The first panel shows the data and a counterfactual prediction for the post-treatment period. The second panel shows the difference between observed data and counterfactual predictions. This is the pointwise causal effect, as estimated by the model. The third panel adds up the pointwise contributions from the second panel, resulting in a plot of the cumulative effect of the intervention.

As presented in Table 1 and Figure 3 below, during the post-intervention period, the average number of Asacol^®^ prescriptions per day was 0.77. In the absence of an intervention, we expected an average of 0.60 with 95% [CI: 0.34, 0.85]. Subtracting the prediction from the observed response yielded an estimate of the causal effect of the intervention on prescriptions of Asacol^®^ at 0.17 with 95% [CI: −0.08, 0.41]. Post-intervention Asacol^®^ was 140.00 prescriptions compared to the predicted 108.65 prescriptions at baseline 95% [CI: 65.22, 154.23].

By contrast, in terms of the relative effect, the prescription of Asacol^®^ showed an increase of +29% over a 95% confidence interval [−14%, +69%], but this change was not statistically significant.

#### 3.1.2. Aggregated Cost Nudge

**Figure 4:**
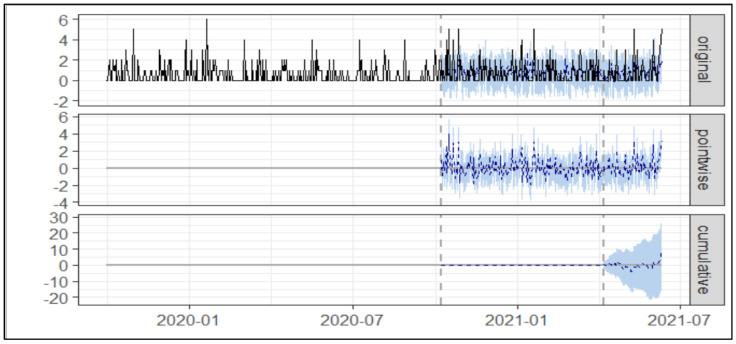
Trend of prescription of Asacol^®^ post intervention 1 and post intervention 2. Note: The first panel shows the data and a counterfactual prediction for the post-treatment period. The second panel shows the difference between observed data and counterfactual predictions. This is the pointwise causal effect, as estimated by the model. The third panel adds up the pointwise contributions from the second panel, resulting in a plot of the cumulative effect of the intervention.

As presented in Table 2 and Figure 4 below, during the post-intervention 2 period, the average number of Asacol^®^ prescriptions was 0.80. In the absence of an intervention, we would have expected an average of 0.67 with 95% [CI:0.36, 1.00].

Table 2 shows the absolute effect of the intervention was 0.13 with a 95% [CI:−0.20, 0.44], and in relative terms of Asacol^®^ prescription increased by +20.00% with 95.00% [CI:−30.00%, +67.00%] suggesting no significant statistical change.

### 3.2. Octasa^®^ Usage

#### 3.2.1. Simple Cost Nudge

**Figure 5:**
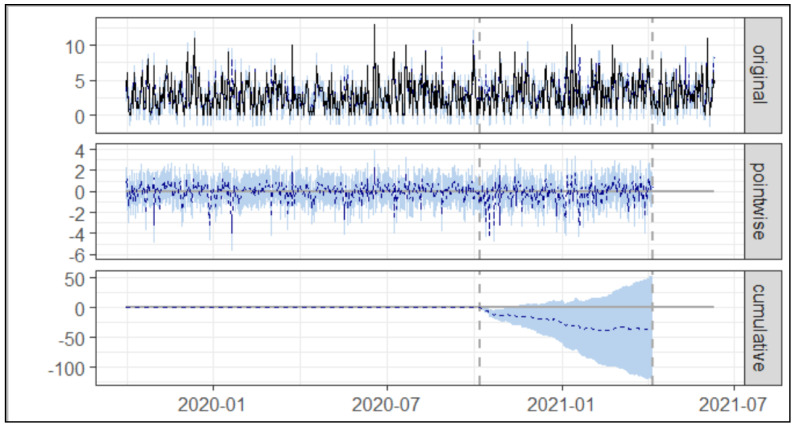
The trend of prescription of Octasa^®^ prior to and during the implementation of intervention 1. Note: The first panel shows the data and a counterfactual prediction for the post-treatment period. The second panel shows the difference between observed data and counterfactual predictions. This is the pointwise causal effect, as estimated by the model. The third panel adds up the pointwise contributions from the second panel, resulting in a plot of the cumulative effect of the intervention.

During the post-intervention period, the average number of Octasa^®^ prescribed per day was 3.22. In the absence of an intervention, we expected the average prescriptions per day to be 3.40 with 95% [CI:2.94, 3.91]. The absolute effect of intervention 1 was found to be −0.18 with a 95% [CI:−0.69, 0.27]. In relative terms, the Octasa^®^ prescription showed a decrease of −5.30% with 95% [CI:−20.00%, +8.10%]. This was a statistically insignificant result. Further details are presented in Table 3 and Figure 5.

#### 3.2.2. Aggregated Cost Nudge

**Figure 6:**
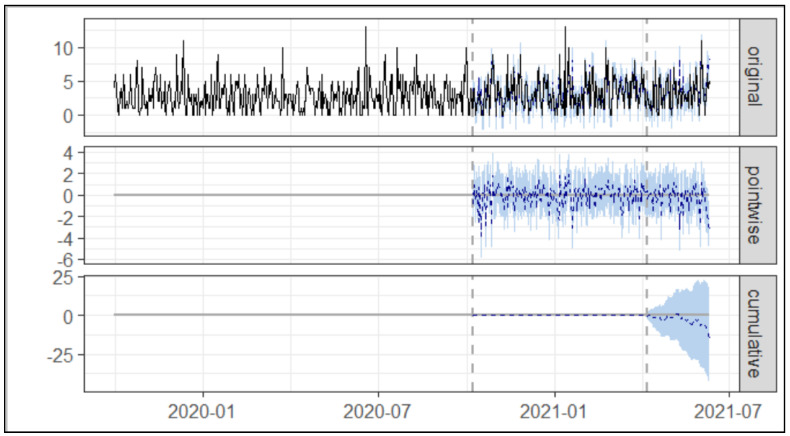
The trend of prescription of Octasa^®^ post intervention 1 and post intervention 2. Note: The first panel shows the data and a counterfactual prediction for the post-treatment period. The second panel shows the difference between observed data and counterfactual predictions. This is the pointwise causal effect, as estimated by the model. The third panel adds up the pointwise contributions from the second panel, resulting in a plot of the cumulative effect of the intervention.

During the post-intervention period, the average number of Octasa^®^ prescribed per day was 3.1. In the absence of an intervention, we would have expected an average response of 3.30 with 95% [CI: 2.94, 3.91]. Subtracting this prediction from the observed response yielded an estimate of the causal effect the intervention had on the prescription of Octasa^®^. This effect is −0.23 with a 95% interval of [−0.69, 0.22]. Further information is available in Table 4 and is illustrated in Figure 6.

In relative terms, the response variable showed a decrease of −7%. The 95% interval of this percentage is [−21.00%, +6.50%], however, this effect is not statistically significant.

## 4. Discussion

The current paper evaluated the impact of two nudges. The nudges were implemented through an electronic prescribing system already in use at the hospital. Both nudges aimed to increase clinicians’ prescriptions of Octasa^®^ relative to Asacol^®^. The first used simple cost information (percentage difference), and the second used aggregated cost information (potential savings for the NHS system). Our results suggest that neither nudge was effective.

Nudges do not always work. A recent systematic review of 15 studies, evaluating 20 different intervention studies found that 20% of the interventions did not work [25]. Nudges are designed to non-coercively influence behaviour without forbidding options [26,27,28,29], and it is the clinicians who must interpret and act. Four potential reasons why our nudges did not change prescribing are explored here. First, when prescribers experience alert fatigue, essentially checking the tick-box to move on before reading, electronic prompts are unlikely to work [30]. Second, reactance theory suggests that even when people do not explicitly realise they are being nudged [31], they may sense a loss of freedom [32]. They may interpret this to mean that someone is trying to control their choices (i.e., Big Brother) and so react negatively. Third, our use of aggregated costs likely made the messages more obscure. It is possible that prescribers who read our messages did not understand them.

A fourth potential reason why our nudges were not effective is that clinicians may not consider costs to be a relevant part of their decision-making [33]. Foremost in a clinician’s mind is making the best clinical decisions for their patients, and not how those decisions economically affect the wider health system. How the mantra “do no harm” extends from individual patients to the wider health system is a topic of heated debate [34], as is how cost-effectiveness should be calculated [35]. However, when two treatments are equally effective clinically but entail different financial costs, then choosing the cheaper treatment should not be controversial. In some situations, the responsibility for this choice could be removed from the clinician and placed with the hospital pharmacy. This said, if no discussion were required between clinicians and pharmacists, this action could be highly contentious [36].

As electronic prescribing becomes more common, nudges implemented through an electronic prescribing system may also become more common. Future formative studies may suggest ways to enhance the effectiveness of nudge message prompts. Based on the above reasons our nudges may not have worked, we suggest that message prompts must (1) avoid falling prey to alert fatigue, (2) retain clinicians’ sense of autonomy, (3) be easy to understand, and (4) provide meaningful information. Future realist evaluations could also be used to reveal what nudges work well, where, and why [37]. These insights for future interventions and research are highly recommended by the authors.

One of the major strengths of this study is the positive relationship we developed with stakeholders. This positive relationship allowed us to identify a meaningful clinical issue and to negotiate how to display cost information without revealing privately negotiated prices. A limitation of the study is its size and scope. As the study involved only one hospital and only one pair of medications, one needs to be very careful about generalising even negative findings. Another limitation is that data were not captured to describe the time prescribers spent viewing the nudges. Since the data provided to the researchers were anonymous, it cannot be confirmed whether the doctors were exposed to both nudges. Doctors might have left and joined the hospital during the study period. We recommend that further studies consider these limitations and where possible expand their plans to collect secondary data.

A final limitation mentioned here is that our nudges were based entirely on cost-effectiveness and not at all on clinical effectiveness and patient safety. For many treatment tradeoffs, this focus could be inappropriate. As the medications in this study were equally clinically effective and safe, this was not a problem for our interventions. However, we assumed clinicians knew these medications were equally effective and safe, and this may have been a problem for our interventions. Guidelines in medicine change rapidly, and, as stated above, foremost in a clinician’s mind is patient health. Where nudge can provide additional information, prompts about effectiveness and safety could help clinicians keep up with rapidly changing guidelines and treatment options.

## 5. Conclusions

While the interventions in the present study were not effective, previous studies have demonstrated that presenting cost information can influence prescribing. Further studies could build on this work to develop more effective nudge intervention prompts. Such research could be complemented by rigorous process evaluations that include quantitative measures (the time prescribers keep the prompt open) and qualitative measures (interviews/workshops). We remain hopeful that environmental modifications that preserve professional choice can positively impact the health economy while maintaining or even increasing care quality.

## Figures and Tables

**Table 1:** Summary statistics—Causal Impact Analysis (pre-intervention 1 vs. intervention 1) for Asacol^®^.

	Average No of Prescription (Asacol^®^)	Cumulative No of Prescription (Asacol^®^)
**Actual**	0.77	140.00
**Prediction (SD)**	0.6 (0.13)	108.6 (23.19)
**95% CI**	[0.34, 0.85]	[62.22, 154.23]
**Absolute effect (SD)**	0.17 (0.13)	31.35 (23.19)
**95% CI**	[−0.08, 0.43]	[−14.23, 77.78]
**Relative effect (SD)**	29% (21%)	29% (21%)
**95% CI**	[−13%, 72%]	[−13%, 72%]

Posterior tail-area probability *p*: 0.096, Posterior prob. of a causal effect: 90%.

**Table 2:** Summary statistics—Causal Impact Analysis (post-intervention 1 vs. post intervention 2) for Asacol^®^.

	Average No of Prescription (Asacol^®^)	Cumulative Number of Prescription (Asacol^®^)
**Actual**	0.80	52.00
**Prediction (SD)**	0.67 (0.17)	43.28 (10.89)
**95% CI**	[0.36, 1.00]	[23.21, 64.86]
**Absolute effect (SD)**	0.13 (0.17)	8.72 (10.89)
**95% CI**	[−0.20, 0.44]	[−12.9, 28.79]
**Relative effect (SD)**	20.00% (25.00%)	20% (25%)
**95% CI**	[−30.00%, 67.00%]	[−30%, 67%]

Posterior tail-area probability *p*: 0.221, Posterior prob. of a causal effect: 78%.

**Table 3:** Summary statistics—Causal Impact Analysis (pre-intervention 1 vs. intervention 1) for Octasa^®^.

	Average No of Prescription (Octasa^®^)	Cumulative No of Prescription (Octasa^®^)
**Actual**	3.22	582.0
**Prediction (SD)**	3.40 (0.24)	614.60 (43.86)
**95% CI**	[2.94, 3.91]	[532.48, 706.87]
**Absolute effect (SD)**	−0.18 (0.24)	−32.60 (43.86)
**95% CI**	[−0.69, 0.27]	[−124.87, 49.52]
**Relative effect (SD)**	−5.30% (7.10%)	−5.30% (7.10%)
**95% CI**	[−20.00%, 8.10%]	[−20.00%, 8.10%]

Posterior tail-area probability *p*: 0.231, Posterior prob. of a causal effect: 77%.

**Table 4:** Summary statistics—Causal Impact Analysis (post-intervention 1 vs. post intervention 2) for Octasa^®^.

	Average No of Prescription (Octasa^®^)	Cumulative No of Prescription (Octasa^®^)
**Actual**	3.10	200.00
**Prediction (SD)**	3.30 (0.24)	215.00 (15.70)
**95% CI**	[2.80, 3.80]	[183.80, 245.50]
**Absolute effect (SD)**	−0.23 (0.24)	−14.96 (15.62)
**95% CI**	[−0.69, 0.22]	[−45.08, 14.00]
**Relative effect (SD)**	−7.00% (7.30%)	−7.00% (7.30%)
**95% CI**	[−21.00%, 6.50%]	[−21.00%, 6.50%]

Posterior tail-area probability *p*: 0.162, Posterior prob. of a causal effect: 84%.

## Data Availability

The raw data supporting the conclusions of this article will be made available by the authors without undue reservation.
